# “The ban is there, but it is not there”: perceptions of cigarette users and tobacco vendors regarding ban on the sale of loose cigarettes in India

**DOI:** 10.3389/fpubh.2024.1375113

**Published:** 2024-05-30

**Authors:** Mayank Sakhuja, Mark M. Macauda, James F. Thrasher, James R. Hebert, Mangesh S. Pednekar, Prakash C. Gupta, Daniela B. Friedman

**Affiliations:** ^1^Department of Health Promotion, Education, and Behavior, Arnold School of Public Health, University of South Carolina, Columbia, SC, United States; ^2^UNC Lineberger Comprehensive Cancer Center, University of North Carolina at Chapel Hill, Chapel Hill, NC, United States; ^3^Department of Epidemiology and Biostatistics and Cancer Prevention and Control Program, Arnold School of Public Health, University of South Carolina, Columbia, SC, United States; ^4^Healis Sekhsaria Institute for Public Health, Navi Mumbai, India

**Keywords:** tobacco control, loose cigarettes, singles, India, tobacco policy, Qualitative research, Stakeholder analysis

## Abstract

**Introduction:**

Banning the sales of loose cigarettes is recommended by Article 16 of the World Health Organization – Framework Convention on Tobacco Control. This study aims to understand the perceptions of cigarette users and tobacco vendors regarding such a ban.

**Methods:**

Using a systematic recruitment and interview protocol, we interviewed cigarette users (*n* = 28) and tobacco vendors (*n* = 28) from two Indian cities where sales of loose cigarettes were banned (Mumbai) or not banned (Delhi). Separate semi-structured interview guides were used for users and vendors. Interview questions focused on reasons for purchasing loose cigarettes, preference for buying and selling loose vs. packs, thoughts on the necessity of banning loose cigarettes, and the perceived impact of the policy ban for vendors and cigarette users. We performed thematic analysis and used NVivo for organizing transcript coding.

**Results:**

The main reasons users cited for purchasing loose cigarettes were financial constraints, social restrictions (fear of getting caught), and limiting cigarette consumption. In Mumbai, awareness of the existing ban was poor among both users and vendors. Those who were aware did not think the policy had been implemented. Users thought that loose cigarettes promoted smoking initiation and prevented them from quitting. Both users and vendors reported that a ban on loose cigarettes would reduce cigarette consumption and promote quit attempts as it would not be possible for everyone to purchase packs because of financial and social reasons.

**Conclusion:**

Users in both cities reported easy access to and widespread availability of loose cigarettes. Low awareness of the ban in Mumbai suggested inadequate enforcement. A country-wide ban on the sale of loose cigarettes could be highly effective in preventing smoking initiation and promoting quitting.

## Introduction

Tobacco consumption is attributed to 1.3 million deaths each year in India (1, 2) and about 11% of Indian population aged 15 years and above are current tobacco smokers (3). Despite adoption of significant tobacco control policies in India (4), the availability of loose cigarette sale in both regulated and unregulated Indian markets appears to promote the tobacco epidemic in the country (5, 6).

The majority of cigarettes sold and purchased in India are in the loose form (7). According to the 2016–17 Global Adult Tobacco Survey (GATS) in India, about 67% of cigarette users reported having purchased loose cigarettes at their last purchase (3). Most tobacco vendors in India continue to sell loose cigarettes that are removed from their commercially packed cigarette boxes. A study that estimated the sale of loose cigarettes found that about 75% of total cigarettes sold in 10 jurisdictions, representing the four regions of India, were in loose (8). Another cross-sectional study based in India found that 95.5% of the tobacco vendors reported selling loose cigarettes and that loosies were most frequently purchased by adult men and college students (9). Similarly, Goel et al. (10) found that 93% of the tobacco vendors were selling loose cigarettes and most of them were located in urban neighborhoods and sold tobacco products to minors.

The availability of loose cigarettes is significantly associated with the sale of tobacco products to minors (11), thus potentially creating a gateway to addiction. Smokers perceived loose cigarettes to be more affordable (per purchase) compared to the cost of the whole cigarette pack (12, 13). Studies conducted among adult Mexican smokers found that a sight of loose cigarette sale acted as a cue to smoking (14). Also, the sales of loose cigarettes in smokers’ neighborhoods was associated with smokers reporting cravings to smoke, which was found to be positively associated with the purchase and consumption of loose cigarettes (15).

The sales of loose cigarettes in India goes against Article 16 of the World Health Organization Framework Convention on Tobacco Control (WHO-FCTC) that recommends banning the sale of loose cigarettes (16). Additionally, loose cigarettes also violate several central legislations in India. For example, the Legal Metrology Act, 2009 prohibits sale of products without their commercial packaging, and Section 7 of the Cigarettes and Other Tobacco Products Act (COTPA), 2003 that requires 85% area of the cigarette packs be covered with health warning labels (17). As per the proposed amendment to the COTPA in 2020, cigarettes/bidis and other tobacco products must be sold and purchased in their sealed, intact, and original packaging (17).

Following these amendments, many Indian states have banned loose cigarette sales (6, 18–21). Many of the remaining states that represent densely populated regions with high smoking prevalence have not yet implemented the ban. As per the recommendations of the Parliamentary Standing Committee on Health and Family Welfare to the Government of India in December 2022, India is now considering a national ban on the sale of loose cigarettes (22, 23). With the ongoing advocacy efforts to ban loose cigarette sales in India, it becomes important to understand perceptions of key stakeholders regarding a ban on loose cigarette sales and generate preliminary evidence for policy effectiveness and implementation as has been examined globally for various tobacco control policies such as the nicotine reduction policy (24) menthol cigarette ban regulation (25), and risk messaging about tobacco constituents (26) in the United States. This study is therefore very timely and novel, as it aimed to evaluate perceptions of cigarette users and tobacco vendors regarding the ban on loose cigarette sales in two Indian cities, Mumbai, where the ban was already implemented, and Delhi, where the ban was not implemented – to not only evaluate the existing policy but also to generate evidence on how loose cigarette sales ban affect smoking behavior that will guide the implementation efforts across country.

## Methods

### Study setting

Two major urban cities were selected, one where sale of loose cigarette was banned (Mumbai), and one where sale of loose cigarette was not banned (Delhi). Within each city, based on discussions with in-country partners, we selected economically and socially diverse neighborhoods. Within each identified neighborhood, we further identified more distinctive neighborhoods such as tourist places, university areas, urban villages, shopping malls, prominent landmarks, public or private schools, metro stations, shopping complexes, prominent government buildings, hospitals, and commercial office places, where participants were recruited and interviewed.

### Recruitment protocol for tobacco vendors

To recruit tobacco vendors who sold loose cigarettes and/or loose bidis in Mumbai and Delhi, a systematic protocol was followed. The protocol was developed by leveraging insights from established field protocols that have been used in India to evaluate health warning compliance of tobacco products (27, 28). Subsequently, the protocol was tailored and refined to suit the specific needs of this study based on discussions with our in-country partners. Our plan was to interview four different types of tobacco vendors: (a) permanent tobacco shops, (b) small tobacco kiosks, (c) street vendors, and (d) grocery stores (see Table 1 for definitions). Within each distinctive neighborhood, we aimed to identify, recruit, and interview at least one vendor of each of the four types. MS, who was the primary data collector, visited the identified distinct neighborhood. On the first visit, MS explored the neighborhood by foot or by public/private transport to understand the area better and would also speak with locals like shop owners, pedestrians, or drivers of public transport to learn more about the neighborhood and the areas where commercial activities took place within and around that distinct neighborhood. On the next visit to that neighborhood, MS would arrive at a randomly chosen fixed point. From that fixed point, MS walked toward the area of commercial activity where multiple shops were present.

**Table 1:** Operational definitions of tobacco vendors.

Vendor type	Definition
Permanent tobacco shops	A shop enclosed in a permanent building structure which sells multiple types of tobacco products.
Small tobacco kiosks	A small shop, which is not enclosed in a permanent structure, rather built like a small booth made of aluminum/steel/tin with an open window, which sells cigarettes, bidis, pan masala, paan, candies, and mouth fresheners.
Street vendor	An individual who does not have a permanent place of business or an establishment, and primarily sells cigarettes, bidis, and pan masala on streets on a moving cart/vehicle or on foot.
Grocery stores	A shop enclosed in a permanent building structure which sells grocery items, general items, cleaning supplies, and tobacco products.

Facing toward the area of commercial activity, MS would first look for a permanent tobacco shop. If a permanent tobacco shop was present, MS approached the first available shop. Before initiating any discussion, MS observed from a distance, transactions between the vendor and customers. Observation was done to determine if the vendor was selling loose cigarettes or loose bidis to its customers, which was the inclusion criteria for tobacco vendors. Once inclusion criteria was met, MS followed the interview protocol described in the next section.

If that specific vendor did not agree to participate, MS looked for another nearest permanent tobacco shop within the same commercial area and approached the vendor in the same way as described above. If none of the permanent tobacco shop vendors agreed to participate, MS then looked for small tobacco kiosks within the same commercial area.

If a small tobacco kiosk was available, then MS would approach the vendor in the same way as described above, and if not, then MS would look for grocery store owners in the same commercial area. If no grocery store was found in the commercial area, then MS also traveled to a nearby residential area since grocery stores are usually located around residential colonies.

Since street vendors do not have a permanent location to do business and do not have a proper establishment like other vendor types, MS looked out for street vendors in all neighborhoods he visited. If any street vendor was identified and any other type of vendor was already interviewed from that neighborhood, MS still approached the street vendor for the interview since it was difficult to locate them because of their continuous mobility or small establishment structure.

If none of the vendor types were able to be interviewed, MS then traveled toward the other potential commercial area within that neighborhood. On reaching the second commercial area, MS would look for the first available permanent tobacco shop (if permanent tobacco shop vendor was not interviewed in the previous commercial area). However, if a permanent tobacco shop vendor was interviewed in the first commercial area, then MS looked for a small tobacco kiosk in the second commercial area. If a small tobacco kiosk was not found, MS then looked for a grocery store. MS followed this process until all four vendor types were interviewed or MS had covered four commercial areas in the identified distinct neighborhood. A similar process was followed in other distinct neighborhoods as well.

### Interview protocol for tobacco vendors

If it was observed that the vendor sold loose cigarettes and/or loose bidis, MS would then approach the vendor and spend considerable time building rapport before introducing the research project. Once MS felt that the vendor was not hesitant in continuing the conversation, he then introduced himself as a doctoral student working on a research project focused on loose cigarettes. MS would then describe the purpose of the study and asked the vendor if he would like to participate in a brief conversation on the topic. If the vendor agreed, then he was asked for a suitable time to have a conversation. Those who agreed to participate either spoke at the same time or asked MS to come at a specific time depending on their availability and customer load. Before starting the interview, MS completed a brief quantitative survey described in the following section. All interviews were conducted in-person and outdoor at the vendor’s shop and were audio-recorded after receiving consent from the vendor. Once the interview was over and participants were offered a small financial incentive for their time, MS himself completed an observational checklist described later. Finally, MS took pictures of the vendor’s shop after getting permission from the vendor.

### Survey instrument for tobacco vendors

The brief survey instrument included questions about vendor’s age, type of store, and buying behavior of customers separately for cigarettes and bidis with five response options (only singles/mostly singles/singles and packs equally/mostly packs/only packs). Vendors were also asked, “Of 100 daily customers, how many of them visited to purchase singles.”

### Observational checklist for tobacco vendors

After the interview, MS observed and noted other items being sold by the vendor; whether tobacco products were displayed and if yes, whether pictorial warnings on those displayed products were clearly visible from the entrance of the shop; availability of smoking aids such as lighters, ashtrays; whether statutory warnings were present and if they clearly depicted the ill effects of tobacco; and whether the vendor advertised any tobacco product.

### Recruitment protocol for smokers

Loose cigarette/bidi smokers were identified primarily at the tobacco shops that were visited for recruiting vendors. After completing the recruitment and interview procedure with the vendor, MS would observe customers who bought loose cigarettes/bidis from the vendor. If any customer purchased a loose cigarette/bidi and smoked near the vendor’s shop, MS would then approach the customer and introduce himself. MS then described the purpose of the research project and invited the individual for a brief interview. MS assured the individual it would be a brief conversation and that they did not need to answer any question with which they felt uncomfortable. Some agreed to be interviewed on the spot whereas others who could not participate immediately shared their contact number or email and asked to schedule a later time for the interview. They were later reached out again via call or email to determine their availability.

In addition to recruiting smokers from tobacco shops, we also used a snowball sampling approach (29) where MS after having interviewed a participant asked them to recommend others, they knew who smoked cigarettes and/or bidis. Recommended participants were later contacted and asked if they had purchased loose cigarette/bidi at their last purchase or in the last 30 days, to determine if they met the inclusion criteria.

### Interview protocol for smokers

Smokers who agreed to participate on the spot were interviewed outdoors, somewhere near the vendor’s shop where they were initially approached. Interviews with others, who requested to schedule some other time and connect virtually, were conducted online, via zoom. After obtaining consent, all interviews were audio-recorded, and all participants were offered a small financial incentive after the interview. Prior to the interview, participants answered a brief survey focused on capturing their socio-demographic information and their tobacco use status, such as smoking frequency, and quit intentions.

### Data collection tools

Two semi-structured interview guides were developed for conducting interviews with smokers and vendors. Interview questions for smokers primarily focused on understanding reasons for purchasing loose cigarettes/bidis, awareness regarding the ban on the sale of loose cigarettes and policy implementation status (for smokers in Mumbai), thoughts on the necessity of banning loose cigarettes, and the perceived impact of the policy ban. Interview questions for vendors focused on reasons why individuals purchased loose cigarettes, preference for selling loose/packed cigarettes, awareness regarding the ban on the sale of loose cigarettes and policy implementation status (for vendors in Mumbai), and how would the policy ban impact the vendor, and customer’s buying behavior.

### Data analysis

All audio files were organized in NVivo (30), and reflexive thematic analysis, a widely used qualitative analytic method, was performed that involved reporting the identified and analyzed themes and patterns within the dataset (31). Principles of thematic analysis as outlined by Braun and Clarke (31) guided the analysis. First, all transcripts were checked back against the original audio files to ensure accuracy. A preliminary codebook was then developed using the original interview guide. Three authors (MS, DBF, and MMM) independently coded one transcript each of smoker and vendor and came together for a discussion to further refine the preliminary codebook and added relevant codes. Later, additional codes were added while reviewing every transcript line-by-line (32). Upon coding completion, data was analyzed to identify patterns, and codes were grouped together into meaningful themes for interpretation (33).

## Results

Participant characteristics for cigarette users are presented in Table 2, and participant characteristics for tobacco vendors are presented in Table 3. In the following sections, we present the findings comparing perceptions of cigarette users and tobacco vendors by three major themes: (a) reasons for purchasing loose cigarettes; (b) awareness of the policy and policy implementation status; and (c) impact of the policy on smoking behavior.

**Table 2:** Cigarette users’ characteristics (*N* = 28).

Variable	*N* (%)
City	
Delhi	15 (53.6%)
Mumbai	13 (46.4%)
Sex	
Male	19 (67.9%)
Female	9 (32.1%)
Education	
Low	4 (14.3%)
Moderate	6 (21.4%)
High	18 (64.3%)
Occupation	
Organized sector	12 (42.9%)
Unorganized sector	5 (17.9%)
Unemployed	7 (25%)
Self-employed	4 (14.3%)
Marital status	
Married	4 (14.3%)
Unmarried	23 (82.1%)
Divorced	1 (3.6%)
Age (Mean ± *SD*)	26.4 ± 6.2
Last product purchased	
Cigarette	28 (100%)
Last purchase type	
Loose	24 (85.7%)
Pack	4 (14.3%)
Smoking frequency	
Non-daily	11 (39.3%)
Daily	17 (60.7%)
Tobacco use status	
Exclusive smoked tobacco user	25 (89.3%)
Mixed tobacco user	3 (10.7%)
Quit intentions	
Within the next month	4 (14.3%)
Within the next 6 months	4 (14.3%)
Sometime in the future, beyond 6 months	7 (25%)
Not planning to quit	13 (46.4%)

**Table 3:** Tobacco vendors’ characteristics (*N* = 28).

Variable	*N* (%)
City	
Delhi	13 (46.4%)
Mumbai	15 (53.6%)
Vendor type	
Permanent tobacco shop	10 (35.7%)
Small tobacco kiosk	9 (32.1%)
Grocery store	6 (21.4%)
Street vendor	3 (10.7%)
For cigarettes, do your customers buy	
Only singles	5 (17.9%)
Mostly singles	18 (64.3%)
Singles and packs equally	2 (7.1%)
Mostly packs	3 (10.7%)
Only packs	–
Of 100 daily customers, how many of them visit to purchase loose?	
Mean	76.6
Range	20–95
For bidis, do your customers buy	
Only singles	–
Mostly singles	–
Singles and packs equally	–
Mostly packs	7 (25%)
Only packs	16 (57.1%)
Did not sell bidis	5 (17.9%)
Displayed cigarettes/bidi packs	
Yes	16 (57.1%)
No	12 (42.9%)
Were pictorial warnings on displayed cigarettes/bidis visible?	
Yes	2 (12.5%)
No	14 (87.5%)
Availability of smoking aids (lighter, ashtrays, matchstick)	
Yes	27 (96.4%)
No	1 (3.6%)
Warning board stating “sale of tobacco products to a person below the age of 18 years is a punishable offense”	
Yes	6 (21.4%)
No	22 (78.6%)
Tobacco advertisement at the entrance	
Yes	16 (57.1%)
No	12 (42.9%)
Tobacco advertisement accompanied by warning	
Yes	15 (93.8%)
No	1 (6.2%)

### Findings from interviews with cigarette users and tobacco vendors

#### Reasons for purchasing loose cigarettes

##### Perceptions of cigarette users

We asked participants “how would they rather buy cigarettes – loose or in packs?” Most participants reported that they would prefer purchasing loose cigarettes. Major reasons mentioned for purchasing loose cigarettes were to limit consumption, intending to quit smoking, financial constraints, and social restrictions.

Cigarette users from both the cities mentioned that they purchased loose to moderate their cigarette consumption as they felt that they would smoke more if they purchased and carried a pack with them.

*“I would not like to buy them in whole packs because when you have more, you are going to smoke more. So, the idea behind buying them loose is I would reduce the number because I will have to go out and buy it every single time. So, the effort is more, so I will try not to make that much of effort for cigarettes and not buy an entire pack.”* – Smoker from Delhi.

*“Because…see if I buy a packet, I know that I have it with me and I can smoke it at any point of time. I just have to take it out and smoke. But I have it in loose, you know, if I have like one with me or two with me, I know that…you know… that this will get over and I have to physically go out and get one for me and then smoke it. So, that involves a bit of effort. So, that sort of reduces the tendency of smoking for me.”* – Smoker from Mumbai.

Users from both the cities also mentioned that they purchased in loose because they smoked occasionally; did not get the urge to smoke frequently; or were intending to quit cigarette smoking.

*“I do not really smoke very often, so that does not make any sense to buy a whole packet and keep it. And also, when I want to, I can just get like a loose one. And especially because I have it just once in a month, so it makes more sense.”* – Smoker from Mumbai.

Many cigarette users stated monetary reasons for purchasing loose cigarettes. They mentioned that it was more economical for them per purchase to spend on loose cigarettes than spending on an entire pack and that they did not have enough money to purchase a whole pack.

Finally, users from both the cities described that they purchased loose because they stayed with their families who were unaware of their smoking status or were staying in a hostel where cigarette smoking was prohibited. Some mentioned that cigarette smoking was prohibited at their workplaces or offices because of which they could not keep a pack.

*“And one more major reason is that I do not smoke at home. I cannot. I’m obviously scared of my parents. So, I do not bring back cigarettes home, ever.”* – Smoker from Delhi.

##### Perceptions of tobacco vendors

We asked tobacco vendors “why did customers purchase loose cigarettes?” Main reasons stated by vendors from Delhi and Mumbai were social restrictions (fear of getting caught at home; smoking restrictions in offices), financial constraints (loose cigarettes being affordable compared to the whole pack), and to limit cigarette consumption (perception that they will smoke more if they’ll purchase a pack).

*“According to me, they buy it like this because of money. Because a pack of cigarettes is expensive. While one packet comes for around Rs. 100, a single cigarette comes for Rs. 10. So that is why, people buy single.”* – Grocery store owner from Mumbai.

*“Some are young and smoke without the knowledge of their parents. They cannot take it home, so they buy loose and smoke and then they go.”* – Small tobacco kiosk from Delhi.

*“One more reason to buy loose cigarettes is if they have a pack in the pocket there is a chance that they might smoke more.”* – Small tobacco kiosk from Delhi.

Vendors from both the cities mentioned similar reasons as cigarette users for purchasing loose cigarettes. They stated that some users had limited capacity to smoke, like smoking just one cigarette a day, so they only purchased loose; some could not take packs at home so preferred purchasing loose; while some found purchasing loose to be pocket-friendly and helpful in limiting their consumption. Tobacco vendors also reported that since tobacco shops were spread throughout the country, it offered cigarette users an easy access to loose cigarettes. Table 4 presents the major reasons provided by cigarette users and tobacco vendors for purchasing loose cigarettes by cigarette users.

**Table 4:** Reasons for purchasing loose cigarettes by cigarette users.

Themes	Cigarette users	Tobacco vendors
**Reduce consumption**		
Limit overall consumption	*“I buy them loose because I used to buy them as packets. But my experience is that when you buy packets, you smoke more. So, that’s why I intentionally do not buy packs.” –* Smoker from Delhi	*“People’s mentality is that if they smoke loose cigarettes then they’ll smoke 3 a day, but if they buy a packet then they might smoke 10 cigarettes, that’s why people buy single only.”* – Permanent tobacco shop from Mumbai
Intending to quit smoking	*“It is only sometimes, you know, when it comes in your mind to stop cigarette smoking, so I do not buy a pack and buy loose cigarettes.” –* Smoker from Delhi	
Low smoking frequency	*“I always prefer to buy them loose because it keeps my habit in check as in I do not get the frequent rush of having a cigarette or anytime I am going outside I do not feel like I should smoke a cigarette now or anything like that because I do not have a habit so I always prefer to buy cigarette in loose, preferably one in number because that actually keeps my habit in check.” –* Smoker from Mumbai	*“And those people who have to smoke only one or two cigarettes in the entire day, they buy loose cigarettes for this reason.” –* Grocery store owner from Delhi
**Financial restrictions**		
Economical to spend on loose cigarettes	*“Suppose there is just me and one friend, and we are like okay let us meet for a walk, and we’ll smoke a cigarette, and then we will go, so that is pocket friendly also, because if I buy a packet of cigarette, it will cost me between Rs. 180 to Rs. 200 and if I am buying loose cigarettes, it will cost me like 36 bucks.” –* Smoker from Mumbai	*“One major reason can be that they do not have to give the bulk amount.”* – Permanent tobacco shop from Mumbai
Limited budget	*“We generally make 10 or 20 rupees with which we can easily buy loose cigarettes but to purchase a pack, we would need 100 rupees which we generally do not have. So, buying loose cigarettes is easy for us.” –* Smoker from Delhi	*“This is South campus [University] area. Students have limited money with them. So, the majority of the people will buy the cigarettes by looking at their pockets. Most of them will buy loose itself, they will not buy packs.”* – Small tobacco kiosk from Delhi
**Social restrictions**		
Hide from family/others	*“No one smokes, my parents or my sister or my brother. My cousins smoke. Everyone knows about them but no one knows about me. It is a kind of safety measure [to purchase loose cigarettes].” –* Smoker from Delhi	*“They mostly prefer loose cigarettes because if they buy a pack they might get caught at home. So that’s why they buy a loose cigarette, smoke here and leave.” –* Permanent tobacco shop from Mumbai
Workplace/office restrictions on smoking	*“At work, if checking happens then I will be in a problem if they find cigarettes with me. But at home, I stay alone so there is no problem as such.” –* Smoker from Delhi	*“The reason is they cannot smoke in the office. They will come and smoke on the roadside. That is one reason.”* – Small tobacco kiosk from Mumbai
Smoking restrictions at public places		*“They cannot buy packets because they have problems at home also, in trains also, and traveling also so people mostly use loose.”* – Permanent tobacco shop from Mumbai
Widely and easily available		*“For addiction and also shops are everywhere. Shops are available throughout the country that is the reason people buy loose cigarettes.” –* Street vendor from Mumbai

#### Awareness of the policy and policy implementation status

##### Perceptions of cigarette users

Since Mumbai had adopted the ban on the sale of loose cigarettes, we asked users if they had heard about the ban. We also asked participants in Delhi if they were aware of the policy being implemented anywhere in the country. Only a few participants from Delhi were aware of the ban being implemented in some of the Indian states. Most participants in Mumbai had no knowledge of the ban on the sale of loose cigarettes and stated that they were easily available and accessible throughout the city.

*“No, even if there is any such rule, I do not have any idea, because till date neither anyone has refused me a loose cigarette, nor I have heard of it.”* – Smoker from Mumbai.

Only a few participants were aware of the ban and reported that nobody was following it and that loose cigarettes were widely available everywhere.

*“I got to know about it through my friends and of course in today’s world, whatever happens, be it funny, be it serious, it is on memes everywhere, so yeah I got to know it through that, I read a little about it but then of course, this being India, people do not take anything seriously… initially, it was there like you have to buy a packet* etc.*, vendors were also like that, but then it is back to normal, you can take as much as you want, no need to buy a packet.”* – Smoker from Mumbai.

We mentioned to the users in Mumbai about the policy ban and asked how easy or difficult did they think was to buy loose cigarettes since the policy was declared. Participants stated that they did not think the policy was implemented as buying a loose cigarette was very easy and was widely available at most stores and around educational campuses, and at tourist places in the city.

*“I mean, I did not even know that this was the rule. I mean the only reason that I did not even know was that it is so easily available. Does not seem like there is a ban. It’s very easily available.” – Smoker from Mumbai.*

*“It is as easy as buying a chocolate or a pack of bread.”* – Smoker from Mumbai.

*“I have not been aware about this policy. I mean, you can just go to any shop in Mumbai and ask them for a loose cigarette. Even in Marine Drive if you go, the chaiwallah (tea-seller) is there selling tea and you can just ask them for a cigarette and they will give you a loose cigarette.”* – Smoker from Mumbai.

##### Perceptions of tobacco vendors

None of the tobacco vendors in Delhi were aware of the ban on the sale of loose cigarettes anywhere in the country. Most of the vendors in Mumbai were unaware of the ban on the sale of loose cigarettes and mentioned that there was no such rule being implemented and enforced, and that anyone could sell loose cigarettes in the city.

*“Nothing like that, selling loose is on. The government was about to ban it but did not. They have talked about it 5–6 times but have not banned.”* – Street vendor from Mumbai.

*“No, I have not heard about this. I only got to know about it when you told me that sale of loose cigarettes is banned. I did not know it earlier.”* – Permanent tobacco shop from Mumbai.

A few vendors mentioned that they had heard about the ban, but it was not being implemented. They also mentioned that there were talks that the tobacco companies would start producing cigarette packs of fewer cigarettes, but nothing was implemented.

*“Yes, there was a ban on selling loose cigarettes. Maybe 6–8 months ago. It is still not allowed. I heard on the news that there is a ban on selling loose cigarettes, it was there like 6–8 months ago, but then it was not followed. This happened in between, and the company also mentioned that they will start producing a pack of 3 cigarettes, you have to sell 3 cigarettes, but that also did not work out.”* – Permanent tobacco shop from Mumbai.

*“I just heard about it 4–5 years back, that only packets can be sold and not loose cigarette, but after that nothing happened, loose is openly sold.”* – Street vendor from Mumbai.

#### Perceived impact of the policy

##### Perceptions of cigarette users

Cigarette users from both the cities were asked whether the policy would promote or reduce their smoking consumption and were further probed if they would start purchasing packs, or think about making a quit attempt, or switch to other tobacco products such as cheaper cigarettes or bidis. Users’ responses are presented into three major themes: perceptions regarding purchasing packs; perceptions around switching to other tobacco products; and perceptions about the policy leading to quitting behavior and reduced cigarette consumption.

Some users from Mumbai and Delhi mentioned that those who smoked more frequently or were habitual and could afford to buy a cigarette pack would switch to purchasing packs. They also mentioned that switching to packs would increase their cigarette consumption.

*“Yes, if there is a restriction on loose cigarettes and if I buy a pack and keep it with me then instead of smoking 14 cigarettes a week, which I normally do, I may smoke 20 cigarettes. That means I will consume the complete pack within a week which used to get consumed within 10 days earlier. That may get consumed in 7 days.”* – Smoker from Delhi.

*“Those who are habitual will not mind buying a whole pack of cigarettes because they have their set habits and practices. They know how many cigarettes they want to have.”* – Smoker from Mumbai.

However, users did mention that not everyone would be able to buy a pack due to monetary reasons and social norms and restrictions such as fear of smoking in front of parents or fear of getting caught by parents.

Even though some reported switching to packs, users from both the cities also mentioned that individuals would try to reduce their consumption despite purchasing packs as that would start having a psychological impact on them by making them think about the harmful effects of cigarette smoking and motivate them to gradually change their smoking habits.

*“Because from the conversations I have had and the feelings I have had with smoking, when people have to buy packs, it kind of does hit them. That “Oh my God! I” and if they have a pack in their hand and they realize, “Okay, like I have smoked up the whole pack within a day or 2 days or 3 days.” It does hit them much more and it does affects them more psychologically than when they keep walking to their paan [tobacco] shop and keep having conversations with their friends while they are there and smoke cigarettes and lose count of how much they have actually smoked. So, I think if people are buying packs, it impacts them psychologically more.”* – Smoker from Delhi.

A few users from both the cities also described that they would buy packs and share them among their social circles as the ban was on buying loose and not on pack sharing.

*“I would say that I would just look for my friends with whom I used to go for smoking and just go with them and buy a pack of cigarettes and share it among us. That’s what I would do. Regarding whether it would reduce my habit, I really do not think so. If I want to get it, I can still get it. So, the policy would not affect me to that extent or that much.”* – Smoker from Mumbai.

We probed users whether the ban would make them quit smoking or reduce their cigarette consumption and learned that they perceived that their consumption levels would reduce if loose cigarettes were not available as that would reduce the accessibility to loose cigarettes and in return help them forgo a cigarette.

*“I feel it will reduce the smoking pattern of the people. It will be a hassle for a person to get one cigarette then. He would think before, you know, like should I buy it now? He will be like, you know, forced to buy only when he needs it so badly or he will have to depend on others, basically. He will not be independent in purchasing a loose cigarette. He will have to consider other factors.”* – Smoker from Mumbai.

They also stated that banning loose cigarettes would help them in successfully quitting smoking as they will not be able to afford a whole pack. Some also showed concern that buying a pack would mean harming their own health so they would rather quit. Participants who have been trying to quit smoking mentioned that this policy would assist them in successful quitting.

*“Yes, most likely I will try to quit smoking initially. If I will not be able to do so, then will quit gradually.”* – Smoker from Delhi.

*“If the policy gets enforced today then I will quit tomorrow because I feel that I should not pay so much money for a thing that is injurious to my health. Buy a 100 Rupees box and burn the lungs as well. I do not want that.”* – Smoker from Mumbai.

*“If the government completely bans loose cigarettes, then it will help me in quitting. I keep trying to quit and this will be an additional factor which will help me to quit smoking cigarettes.”* – Smoker from Delhi.

*“It will be very relevant because I am trying to quit and seeing that it will be so difficult to quit, you know, like I would have to consider a lot and I do not think I will buy a whole packet of cigarettes. That is because that’s not my smoking pattern right now and I do not want to increase it.”* – Smoker from Mumbai.

Users from both the cities described that they would rather quit smoking as they were aware of its harmful effects and would not want to increase their consumption by purchasing a pack if loose cigarettes were not available.

Finally, it was perceived that the policy would prevent smoking initiation especially among children as they would then be less likely to notice smoking in open places which attracted young children to smoking. Additionally, due to their limited purchasing power, they would not be able to purchase a whole pack.

*“And it would help people reduce their consumption and it would ensure that we do not have like so many like starters for smoking.”* – Smoker from Delhi.

*“Children have less money. Anyone can buy a 10 Rupee cigarette but to buy a 100–200 Rupees packet will be something big for the children and they will not even think about trying, in case they have to buy a packet.”* – Smoker from Mumbai.

We also inquired whether users would switch to other tobacco products such as bidis or cheaper cigarette packs if loose cigarettes were not available. Most users reported that it is less likely that those who smoked cigarettes would switch to smoking bidis as cigarette smoking was perceived to be a status symbol, whereas bidi was not. Bidis were also considered to be harsher than cigarettes.

*“I doubt that. I doubt that because people who are smoking cigarettes, I do not think they will switch to beedis. Until and unless they are too much of brat [spoiled]. I doubt that.”* – Smoker from Delhi.

*“In your college when you are holding a cigarette in your hand, you think you look like a dude, or you look very smart and all. Most of it is show off part. And that show off is only limited to a cigarette and not to a bidi.”* – Smoker from Delhi.

A few users from Delhi also reported that they would switch to other cheaper cigarettes which would only increase their cigarette consumption.

*“There are cheap cigarettes available too. A packet for INR 40, 60. People will surely smoke.”* – Smoker from Delhi.

Even though some responded that switching to cheaper cigarettes was a possibility, a few others stated that it was unlikely to switch to a different brand of cigarette if one has already acquired the taste of other brand.

Finally, a few users mentioned that they would consider quitting cigarette smoking but would also switch to using e-cigarettes.

*“There are chances. Even I am seeing this trend nowadays in my friends that they are turning to this e-cigarette thing which is also very easily available.”* – Smoker from Mumbai.

##### Perceptions of tobacco vendors

We asked the vendors what changes they have observed (those who were aware of the ban in Mumbai) or anticipate observing in their customer’s buying behavior and smoking patterns. They reported that if loose cigarettes were banned, most people would not purchase packs due to financial constraints.

One vendor from Mumbai described his experience of not selling loose cigarettes for a few days when he heard about the ban on the sale of loose cigarettes. He mentioned that cigarettes were still being sold but his sales got reduced as those who smoked occasionally did not purchase a pack when loose cigarettes were not available.

*“Cigarettes were still being sold but the sales got reduced by 20–30%, because a person who smokes only single will not purchase a pack, so that affected our business.”* – Permanent tobacco shop from Mumbai.

Some vendors from Delhi and Mumbai also reported that users would start purchasing packs. They stated that those who were habitual or highly addicted to smoking and had enough money to buy a pack would switch to purchasing packs and the policy would not impact them much but would rather increase their smoking consumption.

*“All the high class and rich people will buy packets.”* – Permanent tobacco shop from Delhi.

*“The addict will buy a packet and smoke. If he has money, he will continue smoking.”* – Street vendor from Mumbai.

However, when probed, they mentioned that not everyone would be able to purchase packs. Users would think a lot before buying a pack, and they would not buy it if they did not have the capacity to smoke a whole pack.

*“It is difficult that everyone will be able to buy a pack because a person thinks even before buying a single cigarette. So, it is difficult to buy a box. If they do not have the capacity, then they will not buy a packet. They will go away.”* – Small tobacco kiosk from Mumbai.

A few vendors also mentioned the possibility of pack sharing. However, they stated that smoking would reduce as not everyone from the group would always be available for contribution.

Vendors from both the cities reported that such a policy would benefit the upcoming generation as they would not be able to purchase packs which would reduce smoking among younger age groups. They also mentioned that individuals belonging to low SES, and occasional smokers would not be purchasing packs and would eventually quit smoking.

*“It is good for youth; they will stop smoking. This policy, if it comes let us not say for us, but it would be very beneficial for the upcoming generation. Upcoming generation who are smoking under the influence of others, they’ll not do it anymore. Yes, there will be an effect. Students will reduce or stop smoking, they’ll not buy packets, and sales will decrease.”* – Permanent tobacco shop from Mumbai.

*“It will be beneficial for those who smoke occasionally because they will not get loose cigarettes and only packet, so that may make them quit smoking.”* – Permanent tobacco shop from Delhi.

*“The people from the lower strata will get impacted. They cannot invest at one time that they buy a full packet and consume it. If they want to do that then how will they pay for the other things? They do not get that much to pay for it. That is their problem. The people from the lower strata will have difficulty buying. They cannot buy a box for 100 or 120 Rupees and consume it throughout the day. They are not able to do that. Somewhere small people will get impacted because they have to watch their pockets.”* – Small tobacco kiosk from Mumbai.

We later probed the vendors to see if smokers would switch to consuming other tobacco products such as bidis or cheaper cigarettes if loose cigarettes were not available. Most described that it was less likely that smokers would switch because they have got into the habit of smoking a particular brand of cigarette, and that bidis were harsher compared to cigarettes and were preferred only by the labor class or individuals belonging to the lower SES.

*“Very less likely that people will switch to bidis. That is because of the beedi and cigarette, if we consider the filter then the beedi hits more, it is harsher and the cigarette is smooth. So, I do not think that people will prefer beedi.”* – Small tobacco kiosk from Mumbai.

*“They will not smoke bidis. Only the labor and people belonging to low income groups smoke bidis. People who are of higher-class smoke cigarette only.”* – Small tobacco kiosk from Mumbai.

A few vendors also pointed to the possibility that buyers might switch to purchasing cheaper cigarettes which cost less than their usual brand. Table 5 presents the main themes regarding the perceived impact on cigarette users’ buying behavior.

**Table 5:** Perceived impact of the loose cigarette ban on cigarette users’ purchase behavior.

Themes	Cigarette users	Tobacco vendors
**Perceptions regarding purchasing packs**		
Users will switch to purchasing packs.	*“I have friends who smoke 1½ boxes daily. So, they will not get affected by this. If such a policy is implemented in India, sorry in Bombay, that will not make any difference to them because they anyway smoke 1½ cigarette box per day. So, if they do not get loose cigarettes then they will buy a box.”* – Smoker from Mumbai	*“They will buy a box of course because as per habit of those people who smoke, they will purchase a box. Because their habit cannot be stopped immediately. They cannot stop the habit once they have started. It is not possible. They will purchase.” –* Grocery store owner from Mumbai
Users will not switch to purchasing packs because of financial and social restrictions.	*“No, even if I look, consider myself when I was in college, I never used to purchase a pack because I was scared, you know, where will I keep it, what if you know, my mother or my dad catch me, you know…”* – Smoker from Delhi	*“Sales would reduce by 50%. If they will not get loose cigarettes they will not smoke, because they will not have enough money to buy the pack.”* – Permanent tobacco shop from Mumbai
Users will switch to packs but also try to reduce consumption.	*“I will have to change my habit, because if I will not change my habit, then earlier where I was smoking 10 cigarettes, now I will start to buy 10 packets, it will be like that. Because it will then be in my pocket all the time, and I will smoke in every 2 min. So if I will not change my habit, then I would be at loss. If it gets implemented from tomorrow, it will not be possible for me to change my habit all at once at the starting, I will have to change my habits gradually. In the beginning it [smoking] will increase, but smokers will also realize that slowly, if he is sincere… I am not talking about drunkards or heavily addicted, so moving forward they will reduce their consumption on their own. In the beginning, everyone will start smoking more, who used to smoke a single… if he buys a packet, he will smoke more in the beginning, but afterward he will control slowly.”* – Smoker from Mumbai	*“No matter how addicted they are they’ll always try to control. Earlier they were stubborn about smoking but not anymore. Because of the corona, people have changed a lot.”* – Permanent tobacco shop from Mumbai
Users will share packs.	*“See, I can buy a packet and I can share it with my friends, five friends, so I can also contribute with my friends that let us buy a packet of cigarettes and then we will split five in all, like that. So, you cannot. The ban comes on buying loose cigarettes but that does not include sharing the cigarettes. So, I do not think so, it’s going to make any difference.”* – Smoker from Delhi	*“All of them will come together, 4–5, if one packet costs 50 Rupees, then each one will pay 10 Rupees and they will buy and smoke again. That can also happen.”* – Small tobacco kiosk from Mumbai
**Perceptions regarding leading to quitting behavior**		
Users’ will reduce cigarette consumption.	*“So, if something like that happens, it will definitely be a good thing. Because it happens that, if you are traveling, or if you are stepping out of Metro or you are on a bus stand or you step out of an auto rickshaw at some stand, and you find a vendor adjacent to the bus stand or whatever. And you are waiting during that time for say 10 min, so you tend to think that since I am waiting for 10 min, let me smoke a cigarette till then. So that trend will start disappearing gradually. So, I would say that it would be really good for me.”* – Smoker from Delhi	*“It’s easy for them. They will quit easily. Those who smoke for fun or occasionally, they will smoke once a week. If they will not find one in a week, they will plan for another week and gradually, quit smoking.”* – Permanent tobacco shop from Delhi
Users will get assistance in successful quitting.	*“I will quit, because even if I somehow afford a packet, I cannot take it home with me, I cannot enter the home with it (cigarettes). When I was even outside, when I was in Dehradun, I was alone in hostel, then also I never bought a packet, now I am with family, I cannot take it home, I cannot hide it outside, so then I will have to quit.” –* Smoker from Delhi	*“Many people would try to quit. Suppose poor people, he has Rs. 10, he will not be able to smoke because one packet costs Rs. 100, he will not smoke, slowly he has to quit.”* – Street vendor from Mumbai
Potential users will be restricted to initiate smoking.	*“They [youngsters] will not even start, quit will be the question if they start… there will be a major change in like, like in the next 5–7 years, you know, they will see a majority of the people who are not even starting that bad habit.”* – Smoker from Delhi	*“It will become a compulsion or helplessness, you cannot buy a pack and you are not getting it in singles, then there is no option left at all. Then they have to automatically quit thinking about smoking, and the addiction will not happen. It will be very good for new generation.”* – Small tobacco kiosk from Delhi
**Perceptions regarding switching to other tobacco products**		
Users will not switch to bidis.	*“I think for me that will not happen. For some people that I know, especially men in India and in general I think. But I do feel like the middle class, upper middle class or the corporate, you know, going people, I do not think that they will switch to beedis and other tobacco products. Because it’s also like a status thing.”* – Smoker from Delhi	*“The person who is used to smoking cigarettes will smoke cigarettes till the time cigarette stops coming to the market, even if you give the box worth 200 for 500 or 1,000.”* – Small tobacco kiosk from Delhi
Users will switch to cheaper cigarettes.	*“In some other way, people are going to buy it. So, if not this cigarette, I will go for another cheaper cigarette. There are packets of cigarettes available, even in 100 rupees, 150 rupees. There are packets of cigarettes, it’s a small packet even. So, if you know that there is a pack of 10 cigarettes in 80 rupees even. 150 rupees packet is available. I’m going to buy cheap cigarettes but it’s not going to stop the smoking. It’s going to increase the smoking only.”* – Smoker from Delhi	*“Everyone can buy, if not the expensive ones, then the cheap ones like Rs. 40 – Rs. 50 per pack. Earlier he was smoking a cigarette costing Rs. 20, now he’ll ditch that and switch to a pack of cigarette costing Rs. 50 if he cannot buy loose cigarettes. He’ll start smoking a low quality cigarette.”* – Grocery store owner from Delhi
Users will not switch to cheaper cigarettes.	*“If you started smoking with a particular type of cigarette or a particular type of tobacco. You cannot switch it; your body is used to it. I had started with Marlboro. And I have tried all the types of cigarettes, I have not found a permanent replacement for it. I did try those low on nicotine, low on tobacco, the slim ones, but my body is not getting used to it.”* – Smoker from Mumbai	*“I will tell you, my experience. If you come to me for the small Light Mint and I tell you that the small light mint is not available, only Gold Flake is available. You will say, leave it. You will go to another shop. That is because the person likes it and has got into the habit of smoking it. He will not smoke something else. If their brand is not there, and if I tell them that, then they will not buy it. He will go to another shop. He will not smoke the cheaper one.”* – Small tobacco kiosk from Mumbai
Users will switch to e-cigarettes.	*“Um, I think I would consider quitting maybe, but before that, I would maybe try and switch to another product (e-cigarettes). Not a tobacco product. But maybe, I mean, I do use e-cigarettes. So, I’ll just use more of that.”* – Smoker from Mumbai	

## Discussion

This paper focused on understanding in-depth the perceptions of cigarette users and tobacco vendors regarding the policy ban on the sale of loose cigarettes in two cities of India. Study findings offer insights into the reasons for purchasing loose cigarettes, awareness levels of cigarette users and tobacco vendors regarding the ban on the sale of loose cigarettes, and the perceived impact of the policy ban on cigarette users’ buying behavior. We did not find any differences in the findings between the two cities, despite the ban was in place in Mumbai but not in Delhi, as most cigarette users and vendors from Mumbai were unaware of the ban on the sale of loose cigarettes and stated that no such policy was being implemented or enforced in Mumbai, which made findings from both the cities similar.

Both cigarette users and tobacco vendors in both the cities reported that the main reasons for purchasing loose cigarettes were to reduce smoking consumption, financial restrictions, and social restrictions. Participants reported purchasing loose cigarettes to regulate their smoking consumption as they felt that if they bought packs, they would start consuming more cigarettes. Study findings also suggest that occasional smoking, and intending to quit were also related to purchasing loose cigarettes. Our findings are consistent with the global literature as studies have showed that purchase of loose cigarettes is linked to non-daily smoking (34). Thrasher et al. (15) also found that about one-quarter of the Mexican smokers smoked loose cigarettes to cut down their cigarette consumption. It is well established that the prevalence of loose cigarettes makes tobacco affordable and accessible for minors (10). Studies that examined smokers’ perceptions have found that they perceived loose cigarettes to be more affordable than the cost of the whole pack (12, 13). Qualitative studies among African American urban youth smokers in the United States found that those who purchased loose cigarettes cited ‘less expensive’ compared to purchase of a pack as the most common reason for their purchase (12). Our study findings are consistent as participants reported that it was economical to spend on loose cigarettes and those with a low budget could easily purchase a loosie. Finally, participants mentioned that they feared getting caught by their parents which again aligns with the literature that smokers found carrying cigarette packs to be socially unacceptable and purchased loose cigarettes to hide their smoking habits from others (35).

One of the aims of this study was to assess the impact of the policy ban on the sale of loose cigarettes in Mumbai and how the impact differed between the two cities. However, we found that most of the cigarette users and tobacco vendors were not aware of the policy ban and rather stated that no such ban was being implemented or enforced and that loose cigarettes were widely available. These findings related to low awareness are similar to a study conducted by Eshwari et al. (9) that used cross-sectional surveys to examine perception and practices, and awareness regarding the ban on the sale of loose cigarettes among cigarette users and tobacco vendors in Karnataka, a southern Indian state. They found that 95.5% of the tobacco vendors continued selling loose cigarettes; about half of them were aware of the ban; and only a quarter reported that the ban on the sale of loose cigarettes was implemented (9). Similarly, awareness of the ban among cigarette users was found to be low as well (9).

Both users and vendors reported that individuals would not switch to smoking bidis if loose cigarettes were banned as bidis were primarily consumed by individuals from low SES, whereas cigarettes were preferred by high SES groups and was considered a status symbol. Males, older age groups, and those with lower SES are significantly more likely to smoke bidis (36). Cigarettes, on the other hand, are associated with higher SES and sophisticated lifestyles (37). There is a lack of evidence suggesting that those who are used to cigarette smoking would prefer switching to smoking bidis. However, participants mentioned that individuals could switch to purchasing packs of cheaper cigarette brands if loose cigarettes of their usual brand were not available. That transition could be possible due to the unequal taxes imposed on different types of cigarettes (38). Taxes in India vary by the type of tobacco products, and cigarettes are taxed based on their length, with longer cigarettes taxed at a higher rate compared to cigarettes with shorter lengths (38). We recommend that the taxation system should be simplified, and equal taxes should be imposed across all tobacco products. A higher and equal price would prevent users from switching to cheaper cigarettes.

Cigarette users and tobacco vendors described how a ban on the sale of loose cigarettes would impact the buying behavior of cigarette users. Findings were categorized based on perceptions related to switching to buying cigarette packs, leading to quitting behavior, and switching to other tobacco products. We found that cigarette users with high smoking frequency and those who were already purchasing packs would continue to purchase packs and even if they did purchase packs, they would certainly try to control their consumption. However, not everyone would be able to buy packs due to financial and social constraints. Most participants mentioned that the policy would assist users to quit cigarette smoking or reduce their consumption by forgoing a cigarette. Eshwari et al. (9) found similar results from their cross-sectional surveys with 22% users reporting that if loose cigarettes were banned, they would reduce the number of cigarettes they smoked; 16% would think about quitting; and 9.5% would completely give up smoking. Literature suggests that availability of loose cigarettes decreases the likelihood of making quit attempts (39). Hall et al. (40) found that smokers who lived in a neighborhood where loose cigarettes were easily accessible were less likely to make an attempt to quit cigarette smoking and were more likely to switch back to smoking. Additionally, smokers who purchased loose cigarettes with the intention to limit their smoking were not more likely to quit smoking than those who did not purchase single cigarettes with the intention to reduce their cigarette consumption (15). Thus, our study findings align with the global literature as participants felt that the policy ban would help them in successfully quitting cigarettes. We recommend that longitudinal studies are needed to examine how the policy ban on the sale of loose cigarettes would impact smoking behaviors of individuals with a focus on product switching, quit attempts, successful quitting, and relapse. Even though our study provides strong evidence about the effectiveness of the policy in reducing smoking prevalence, we still recommend that online experiments and simulation studies, as has been conducted for evaluating menthol cigarette ban in the United States (41–43), should be conducted to further strengthen the evidence. Finally, future studies should also assess whether users would switch to products other than cheaper cigarettes and bidis, such as vapes, e-cigarettes, hookahs/waterpipe tobacco, and other emerging products.

This study has several limitations. First, findings from this study are based on a hypothetical situation in the sense that since the policy was not properly implemented or enforced in any of the two cities, participants would not have experienced the real impact of the policy. However, a tobacco vendor who was aware of the policy and did not sell loose cigarettes for some time reported a decline in his sale and cigarette consumption which aligns with the responses of both cigarette users and tobacco vendors. Second, since the policy was not implemented, this study could not measure the actual impact of the policy and thus hinders our ability to draw conclusions about real world effects. Third, participants were recruited from urban neighborhoods of two Indian cities, so, study findings cannot be generalized to rural neighborhoods or other parts of the country. Fourth, the sample size of tobacco vendors is not representative as we could not find many street vendors since they came out late at night which was not always a convenient time for recruitment. Finally, the study findings could only be attributed to cigarette users as we did not find individuals who purchased loose bidis.

## Conclusion and recommendations

Main reasons reported by cigarette users and tobacco vendors for purchasing loose cigarettes were to control cigarette consumption, and financial and social restrictions. Loose cigarettes were widely available in both the cities and users reported easy access despite the ban in Mumbai. Awareness regarding the ban on the sale of loose cigarettes was poor among both users and vendors in Mumbai, implying inadequate implementation and enforcement efforts by the local authorities. Further, all participants from Mumbai reported that even if the ban existed, it was not being implemented by the relevant authorities. Finally, our study findings demonstrate a strong support for implementing the ban on the sale of loose cigarettes as it has the potential to improve public health by reducing tobacco use. Even though some users may switch to purchasing packs, especially heavy users and those who do not intend to quit, and some may switch to cheaper cigarette brands, many believed that the ban would be beneficial in reducing their cigarette consumption, assist them in quitting cigarette smoking and further reducing the chances of relapse, and prevent potential users from initiating smoking. Considering the potential benefits of banning loose cigarette sales, we recommend that efforts be made to strengthen implementation and enforcement measures in order to increase compliance with the ban. Strong and sustained advocacy efforts to effectively implement and enforce a country-wide ban on loose cigarette sales are needed. We also recommend developing health communication messaging and advertisements to not only increase awareness about the ban but also to convey the true purpose of the policy which would help address any potential misinformation spread by the tobacco industry and public’s misperceptions about the policy.

## Data availability statement

The datasets will be made available by the corresponding author only upon reasonable request.

## Ethics statement

The studies involving humans were approved by University of South Carolina and Healis Sekhsaria Institute for Public Health. The studies were conducted in accordance with the local legislation and institutional requirements. The ethics committee/institutional review board waived the requirement of written informed consent for participation from the participants or the participants’ legal guardians/next of kin because the study received an exemption from Human Research Subject Regulation. The study was explained to the participants and verbal consent to participate was obtained before the interview. Since the research involved minimal risk, written consent to participate was not required.

## Author contributions

MS: Writing – original draft, Writing – review & editing, Conceptualization, Data curation, Formal analysis, Funding acquisition, Investigation, Methodology. MM: Writing – review & editing, Conceptualization, Formal analysis, Methodology. JT: Writing – review & editing, Conceptualization, Funding acquisition, Methodology. JH: Writing – review & editing, Conceptualization, Methodology. MP: Methodology, Writing – review & editing, Resources. PG: Methodology, Writing – review & editing, Resources. DF: Writing – review & editing, Conceptualization, Formal analysis, Funding acquisition, Methodology, Supervision.
